# Replication – The ugly duckling of science?

**DOI:** 10.3205/zma000999

**Published:** 2015-11-16

**Authors:** Götz Fabry, Martin R. Fischer

**Affiliations:** 1 Albert-Ludwig-Universität Freiburg, Abt. für Med. Psychologie, Freiburg/Brg., Deutschland; 2 GMS Zeitschrift für Medizinische Ausbildung, stellv. Schriftleiter, Erlangen, Deutschland; 3 Klinikum der Ludwig-Maximilians-Universität München, Institut für Didaktik und Ausbildungsforschung in der Medizin, München, Deutschland; 4 GMS Zeitschrift für Medizinische Ausbildung, Schriftleiter, Erlangen, Deutschland

## Editorial

This August “Science” published a much-noticed paper by a collective of authors demonstrating that the results of many studies in the field of psychology could not be replicated [1]. With great methodological effort the Open Science Collaboration that incorporates 270 scientists from all over the world selected 100 up-to-date experimental studies from three top-ranked psychological journals in order to assign them for replication to designated and qualified research groups. In these replication studies material and instruments from the original studies were used and the authors of these studies were also consulted during the preparation phase. The results are sobering: While 97% of the original studies reported significant results only 36% of the replication studies did so. Furthermore, the reported effect sizes of the replication studies were only half as large as the original ones and even when the original data and the replicated data were analyzed conjointly only 68% of the results turned out to be significant.

What do these findings mean? First of all the problem itself is known for a long time and is not restricted to psychology. Quite recently, we witnessed a sometimes heated discussion circled around the question whether too much “research waste” is produced in the biomedical sciences [2]. As a matter of fact, the replication of many studies in this field fails, even if they are published in top-ranked journals. It is estimated that the proportion of non-replicable studies within the biomedical domain is actually larger than in psychology (approximately 75–90%) and even frequently cited studies make no exception [3].

### Replication in the educational sciences

Against this background one must strongly assume that medical education research is neither exempt from this calamity. Despite the fact that no empirical findings exist to know this for sure, there is some evidence suggesting an urgent need for action here too. A recent study published by the Educational Researcher – the organ of the American Educational Research Association (AERA) – inquired how often replication studies are published in the domain of educational sciences and what kind of evidence they provide [4]. An analysis of all studies published over the period of five years in the 100 top-ranked educational science journals revealed that the proportion of replication studies was 0.13% (221 of 164 589) only. Remarkably, almost two thirds of these studies replicated the results of the original studies. This relatively large proportion however is put into perspective by the fact that more than half of the replication studies were published by the same authors who were also responsible for the original studies. When only those studies were analyzed that had no overlap of authors the proportion of succesful replications declined to about 50%. Thus, if the different approaches are taken into account we see approximately the same picture here as in the current publication on psychological studies, which comes as no surprise given the close proximity of both domains. Furthermore, the psychological replication study provides some additional evidence that also in medical education not everything that glitters is gold. Succesful replication there was more likely when the original P values were smaller (i.e. stricter than .05) and the effect sizes were larger. Unfortunately, both conditions are rather hard to find in medical education studies [5]. In addition, succesful replication was less likely when studies used rather complex procedures, which in turn are quite common in educational studies [6]. These findings suggest the alarming assumption that many insights from medical education research would not withstand a more specific inquiry. Thus, do we need more replication studies in medical education? Why are these studies so rare and what has to change [7]?

#### Replication is more than just history repeating

Some might be thinking back here to the latest reviewer comments that critized the submitted manuscript for being not interesting enough and just repeating what is already known. How does this critique align with the demand for more replication studies? To answer that question it is necessary to take a closer look on the function and characteristics of replication studies.

Overall, replication studies are done to verify scientific evidence. In a review article on replication in the social sciences that is definitely worth reading, Schmidt explained this function in greater detail [8]: Controlling for sampling error and chance (e.g. due to selection bias), insufficient internal validity (e.g. due to intervening variables, regression to the mean, testing effects, etc.) and fraud. Furthermore, replication studies can be used to clarify whether the results of a certain study can be generalized to larger or other populations or to test the hypotheses of the primary study. Considering the function of replication studies more specifically is important because the study design is determined by the purpose of the study [8]. This is especially true because a succesful replication study cannot be a simple “clone” of the primary study. On the one hand this would not be possible at all in typical medical education studies (or generally in psychological or social science studies) as they involve individuals as participants as well as researchers and both cannot be identical at two different points in time. On the other hand, an identical copy of the primary study would not make sense because a verification of research evidence usually requires just to replicate the results at a different point in time at a different place by a different person to accumulate evidence in favor of the transferability and generalizability of the effects found in the primary study. Thus, when designing a replication study it is of utmost importance to reflect precisely which aspects should be held identical and which ones are to be changed in order to gain significant and meaningful results. If, for instance, the study aims at controlling for sampling error or chance it will be necessary that variables and context are as identical as possible to the primary study while the study sample will be different. Typically, this happens when the same researcher replicates a survey or an experiment with a different or a larger sample. If, in contrast, a replication study is done because of doubts regarding the internal validity of the primary study, it will be necessary to replicate the intervention or the measuring procedure as exactly as possible while all other context variables will be different. This usually happens when a different researcher repeats a study with a different sample at a different place under different circumstances. Thus, all studies that are primarily controlling for chance, internal validity, fraud or generalizability might be described as *direct replications*, because certain aspects of the experimental and context conditions of the primary study are repeated as precisely as possible [8].

Things are different however, with studies testing hypotheses. These studies explicitely search for alternative experimental or methodological approaches to gain additional evidence to support the respective construct. Thus, these studies might be described as *conceptual replications* [8]. They are especially relevant since they contribute to completing a theory or to a more comprehensive understanding of constructs or concepts [9]. However, a drawback of these studies is that a failure of the replication does not allow to conclude that this is due to flaws or biases in the primary study as these can only be revealed by direct replications [8]. Thus, a meaningful replication of insights that have already been described elsewhere distinguishes itself by the fact that the replication is the a priori aim of the study and – in accordance with that – that its function is carefully considered against the conceptual background and the preexisting evidence.

#### Duckling or Swan?

Is replication then really the ugly duckling of science that looks rather grey and remains so compared to studies that promise innovative insights? Taking the perspective of the individual researcher it seems that the answer is yes since replication studies are unattractive at most regarding publication prospects and frequency of citations. The review regarding the educational sciences mentioned above reported that 43 of the 100 top journals did not publish any replication study. With regard to citation frequency the original studies yielded 31, the replication studies just 5 citations on average (4). Even if this might at least partly be explained by the fact that the replication studies were published some time after the original studies, the difference remains vast.

However, the perspective of the scientific community on replication studies is different. The structure of a paper already reminds us that replication is one of the defining core principles of science. Good scientific papers delineate the background, methods and results transparently so that other researchers can reconstruct the study not just *in sensu* but – at least as a matter of principle – also *in vivo* (whether we live up to this standard remains open, cf. [10]). Current developments in science already take it a step further so that not just the publications are openly accessible but also the underlying primary data by depositing them in designated repositories. By this means, they can be reviewed independently anytime. This is also possible for publications in our journal because as a part of the platform GMS we are a member of Dryad, an international repository for research data (more information cf. [11]). Against this background replication studies are the touchstone that determines whether the noble principles of science withstand a tangible reality check.

While innovations illustrate what might be possible, replications point out what is likely or valid. Scientific progress needs both. Thus, we should not wait any longer to turn the replication duckling into a swan – and that also holds true for medical education research. However, to facilitate this, the incentives for publishing sound replication studies must change. The GMS Journal for Medical Education will contribute to this development by providing a forum for such studies.

## Competing interests

The authors declare, that they have no competing interests.
